# Physical activity, cardiorespiratory fitness, and metabolic syndrome in adolescents: A cross-sectional study

**DOI:** 10.1186/1471-2458-11-674

**Published:** 2011-08-30

**Authors:** Antonio Stabelini Neto, Jeffer E Sasaki, Luis PG Mascarenhas, Margaret CS Boguszewski, Rodrigo Bozza, Anderson Z Ulbrich, Sergio G da Silva, Wagner de Campos

**Affiliations:** 1 Center for Health Sciences, Universidade Estadual do Norte do Paraná, Jacarezinho, Brazil; 2 Deparment of Kinesiology, University of Massachusetts, Amherst, USA; 3 Departament of Pediatrics, Universidade Federal do Paraná, Curitiba, Brazil; 4 Departament of Physical Education, Universidade Federal do Paraná, Curitiba, Brazil

## Abstract

**Background:**

In adults, there is a substantial body of evidence that physical inactivity or low cardiorespiratory fitness levels are strongly associated with the development of metabolic syndrome. Although this association has been studied extensively in adults, little is known regarding this association in adolescents. The aim of this study was to analyze the association between physical activity and cardiorespiratory fitness levels with metabolic syndrome in Brazilian adolescents.

**Methods:**

A random sample of 223 girls (mean age, 14.4 ± 1.6 years) and 233 boys (mean age, 14.6 ± 1.6 years) was selected for the study. The level of physical activity was determined by the Bouchard three-day physical activity record. Cardiorespiratory fitness was estimated by the Leger 20-meter shuttle run test. The metabolic syndrome components assessed included waist circumference, blood pressure, HDL-cholesterol, triglycerides, and fasting plasma glucose levels. Independent Student *t*-tests were used to assess gender differences. The associations between physical activity and cardiorespiratory fitness with the presence of metabolic syndrome were calculated using logistic regression models adjusted for age and gender.

**Results:**

A high prevalence of metabolic syndrome was observed in inactive adolescents (males, 11.4%; females, 7.2%) and adolescents with low cardiorespiratory fitness levels (males, 13.9%; females, 8.6%). A significant relationship existed between metabolic syndrome and low cardiorespiratory fitness (OR, 3.0 [1.13-7.94]).

**Conclusion:**

The prevalence of metabolic syndrome is high among adolescents who are inactive and those with low cardiorespiratory fitness. Prevention strategies for metabolic syndrome should concentrate on enhancing fitness levels early in life.

## Background

The term metabolic syndrome (MetS) refers to a clustering of cardiovascular risk factors represented by high blood pressure, overweight/obesity, hypertriglyceridemia, low high-density lipoprotein-cholesterol (HDL-C), and glucose intolerance. The diagnosis of MetS in adults, and recently in children and adolescents, is established when three or more of the five individual elements exist together in the same subject [1,2].

Various diagnostic criteria have been proposed by different organizations over the past decade. To standardize the MetS definition, a recent joint scientific statement [3] proposed specific criteria for the clinical diagnosis of MetS in adults. However, there was no consensus regarding the diagnostic criteria for MetS in the pediatric population. Therefore, diagnostic criteria used in adults have been adapted for children and adolescents [4]. These criteria are based on reference values from the NCEP Pediatric Panel report, the American Diabetes Association statement on type 2 diabetes in children and adolescents, and the updated Task Force report on the diagnosis and management of hypertension in childhood [4].

In adults, MetS is associated with a significantly elevated risk of coronary heart disease [5] and diabetes mellitus [6], while in children and adolescents there is a direct relationship between the number of cardiovascular risk factors and the severity of asymptomatic atherosclerosis [7]. There is a substantial body of evidence associating physical inactivity or low cardiorespiratory fitness with the development of MetS in adults [8-10]. This association in children and adolescents is controversial.

However, evidence suggests that sedentary behavior, low levels of physical activity, and cardiorespiratory fitness in youth track into adulthood [11]. Similarly, metabolic risk factors also appear to track over time [12], and may predispose young people to disease later in life [13].

Based on this information, the aim of this study was to estimate the prevalence of MetS and to analyze the association between physical activity and cardiorespiratory fitness with MetS in a random sample of Brazilian adolescents.

## Methods

### Sample

This cross-sectional study was carried out in Curitiba, Paraná, Brazil. The city of Curitiba has a population of 1,678,965 inhabitants with a human development index of 0.763. The sample size consisted of school children registered in the education system (approximately 45,000 students). The following parameters were used to estimate the sample size: an error of 5%; an estimated MetS prevalence of 20%; a design effect of 1.5; a 95% confidence interval; and an additional 10% for losses and refusals. A conglomerate sample of 456 adolescents (223 girls [49%] and 233 boys [51%]) was evaluated. The schools were randomly selected, and the proportion of students was established according to the number of students in each of the nine administrative areas of the city (Santa Felicidade 6.6%; Matriz, 12.3%; Boa Vista, 14.7%; Cajuru, 12%; Portão, 10.6%; Boqueirão, 13.1%; Bairro Novo, 9.6%; Pinheirinho, 9.5%; and CIC, 11.6%). Data collection took place between April and November 2009.

All subjects completed a physical activity questionnaire. Height, weight, blood pressure, cardiorespiratory fitness, and lipid profiles were measured in all subjects. Only students between 10 and < 18 years of age were included in the analyses. Exclusion criteria were the known presence of diabetes and the use of medications that alters blood pressure, glucose, or lipid metabolism.

Written informed consent was obtained from the parent or legal guardian of the adolescent after being given a detailed written explanation of the aims of the study, and the possible hazards, discomfort, and inconvenience. All subjects were given the option to drop out at any time without consequence. This research was approved by the Ethics Committee of the Federal University of Paraná (Resolution 196/96). All procedures and methods in this study conformed to the ethical guidelines established by the World Medical Association's Declaration of Helsinki and the subsequent revisions.

#### Anthropometric measures

Physical measurements were obtained by trained research assistants after blood sample was taken. The body height was measured without shoes to the nearest 0.1 cm with a transportable stadiometer (Ottoboni HM-210D; Ottoboni, Rio de Janeiro, RJ, Brazil). Body weight was measured in light clothing to the nearest 0.1 kg with a calibrated beam balance scale (Toledo 2096 PP; Toledo do Brasil, São Bernardo do Campo, SP, Brazil). Waist circumference was measured at the end of gentle expiration, midway between the lower rib margin and the iliac crest.

#### Blood pressure

Blood pressure (BP) was measured by trained technicians before the blood sampling procedure. The BP measurement was performed according to the method described in *The Fourth Report on the Diagnosis, Evaluation, and Treatment of High Blood Pressure in Children and Adolescents *[14]. The students were asked to rest in the sitting position for 5-10 min prior to the BP assessment. The systolic blood pressure (SBP) and diastolic blood pressure (DBP) were measured in the right arm using a sphygmomanometer of mercury column. Two measurements were obtained after 5 and 10 min of rest. The mean of the two measurements was considered. If the two measurements differed by > 2 mmHg, a third measure was obtained.

#### Blood sampling

Blood samples were obtained by trained and certified nurses between 8:00 and 9:00 AM following an overnight fast. After the blood samples were collected, the subjects were served breakfast, then continued with the physical and fitness measurements. The samples were immediately centrifuged and the serum or plasma separated and placed on dry ice for shipment back to the chemistry laboratory. A single certified laboratory was used for all analyses. HDL-C, triglycerides, and glucose levels were analyzed by colorimetric assay on a random-access Spectrum CCX analyzer (Abbott Diagnostics, Abbott Park, IL, USA).

### MetS definitions

We used the age-modified standards of the ATP III MetS criteria published previously [4] and utilized in others research [15,16]. Abdominal obesity was considered as a waist circumference ≥ 90^th ^percentile for age and gender [17]. High blood pressure was considered as a SBP and/or DBP > 90^th ^percentile by age, gender, and height based on published reference data [14]. Adolescents were considered to have excessive total triglycerides levels if blood concentrations were ≥ 110 mg/dL. HDL-C levels were considered low at a level of ≤ 40 mg/dL, while fasting blood glucose levels ≥ 110 mg/dL were considered indicative of hyperglycemia [4]. MetS was diagnosed when three or more of the five individual elements were present together in the same individual.

#### Physical activity

The Three-Day Physical Activity Record [18] was used to self-record daily activities over 3 days (2 weekdays and 1 weekend day). The daily activities were recorded by adolescents in 15-min segments throughout the day on a scale divided into 9 categories of physical activity (1-2, sedentary activity [lying down and seated]; 3-5, light activity [taking a shower, cooking, taking a walk, and light manual work]; 6-8, moderate activity [sports or leisure activities and moderate manual work; 9, vigorous activity [intense manual work and intense sport or leisure activities]). In this self-report instrument, the subject fills in the boxes corresponding to physical activities performed during the specific time periods.

The questionnaires were self-completed in the classroom with orientation from the researcher. The time spent doing exercises with moderate-to-vigorous intensity (≥ 6 in a physical activity scale) was recorded, then used to determine the total moderate-to-vigorous physical activity (MVPA) expressed in minutes per day. The mean value from the 3 days was considered for the analysis.

The reproducibility of this instrument was reported to be r = 0.91 in subjects from 10 years of age [18], and was validated in adolescents using the technique of double-labeled water [19].

#### Cardiorespiratory fitness

Cardiorespiratory fitness was estimated using the 20-meter shuttle run test, as described by Leger et al. [20]. The test was carried out in a gym with a plane surface. The required speed was continuously increased every minute by 0.5 km/h. Subjects kept the required speed by completing every 20-meter stage within the sound of two beep sounds. The interval between these beeps was reduced every minute in order to elicit the speed increments. The velocity in the last stage completed by each subject was recorded and used to calculate the VO_2máx _in ml.kg^-1^min^-1 ^according to the equation validated by Léger et al. [20], as follows: VO_2máx _= 31.025 + 3.238*velocity - 3.248*age + 0.1536*velocity*age. The authors reported a relationship to directly measured VO_2máx _of 0.84 and a test-retest correlation of 0.98.

### Statistical procedures

Descriptive data are shown as the mean and standard deviation. The Kolmogorov-Smirnov test was used to verify the normality of the physical activity and physical fitness distribution data. To compare the continuous variables between the genders we used Student's *t*-test, and the chi-squared test was used for categorical variables.

The prevalence of MetS and its components was calculated with respect to different cardiorespiratory fitness and physical activity categories. Fitness levels were determined based on tertiles of cardiorespiratory fitness (boys: low < 46 ml.kg^-1^min^-1^, moderate 46-51 ml.kg^-1^min^-1^, and high > 51 ml.kg^-1^min^-1^; girls: low < 38 ml.kg^-1^min^-1^, moderate 38-43 ml.kg^-1^min^-1^, and high > 43 ml.kg^-1^min^-1^. For physical activity, the thresholds were as follows: inactive < 60 min.day of MVPA; active: ≥ 60 and < 90 min/day of MVPA; very active: ≥ 90 min/day of MVPA.

The associations between physical activity and cardiorespiratory fitness with the diagnosis of MetS were calculated using binary logistic regressions. The outcome variable was the presence of MetS. The exposure variables were: participation in moderate-to-vigorous physical activity (inactive, active, and very active) and VO_2max _tertiles (low, moderate, and high). The logistic regression models were adjusted for age and gender. The statistical analyses were performed using SPSS (version 15.0 for Windows; Chicago, IL, USA) [21]. A *p *value < 0.05 denoted statistical significance.

## Results

Descriptive data by gender are shown in Table 1. Boys had higher levels of height, weight, waist circumference, cardiorespiratory fitness, physical activity, SBP, and glucose than girls (p < 0.0001 for all), whereas girls had higher HDL-C values. No differences in age, DBP, and triglycerides levels were observed between genders. The prevalence of MetS was 7.7% among Brazilian adolescents. The prevalence of MetS in boys (10.2%) was significantly higher than in girls (5%; p < 0.05) (Data not shown).

**Table 1:** Anthropometric, cardiorespiratory fitness, physical activity, and individual risk factors characteristics of Brazilian adolescents by gender

	Males N = 233		Females N = 223		p*
	
	Mean	SD	Mean	SD	
Age (years)	14.6	1.6	14.4	1.6	ns
Height (cm)	167.1	11.6	158.8	7.5	< 0.0001
Weight (kg)	57.1	12.5	51.7	10.8	< 0.0001
Waist circumference (cm)	70.8	8.9	65.5	7.0	< 0.0001
VO_2_máx (ml^.-1^kg^.-1^min^-1^)	49.0	6.2	40.5	4.7	< 0.0001
MVPA	136.8	103.3	75.1	82.2	< 0.0001
SBP (mmHg)	100.7	13.9	95.1	13.2	< 0.0001
DBP (mmHg)	69.1	10.6	68.1	10.6	ns
HDL-C (mg/dL)	45.2	11.0	49.9	14.1	< 0.0001
Triglycerides (mg/dL)	89.3	41.3	85.1	34.4	ns
Fasting glucose (mg/dL)	89.4	13.6	83.7	13.8	< 0.0001

Data are means (SD), * Student's *t-test *for differences between gender

Abbreviations: MVPA, Moderate-Vigorous Physical Activity; SBP, Systolic blood pressure; DBP, Diastolic blood pressure; ns, non-significant.

The low HDL-C (33.3%) was the most prevalent component of the MetS criteria, followed by high blood pressure (21.5%), hypertriglyceridemia (19.8%), and hyperglycemia (5.5%) (Data not shown). The prevalence of individual risk factors by gender is shown in Table 2. The prevalence of hyperglycemia and low HDL-C in boys was significantly higher than girls. The proportion of subjects who had 1 or more individual risk factors was 77.1% (Data not shown).

**Table 2:** Distribution of individual risk factors among Brazilian adolescents by gender

	Males N = 233		Females N = 223		p*
	
	%	95% CI	%	95% CI	
Large waist circumference	10.5	7 - 28.5	10.9	7.3 - 15.5	ns
High blood pressure	22.6	17.8 - 28.5	20.3	15.4 - 25.9	ns
Low HDL-C	39.2	33 - 45.4	27	21.5 - 33	0.006
Hypertriglyceridemia	19.8	15.1 - 25.3	19.8	15 - 25.4	ns
Hyperglycemia	7.8	4.9 - 11.8	3.2	1.5 - 6.3	0.033

*Chi-squared test

Abbreviations: 95% IC, Confidence interval of 95%; ns, non-significant.

**Figure 1:**
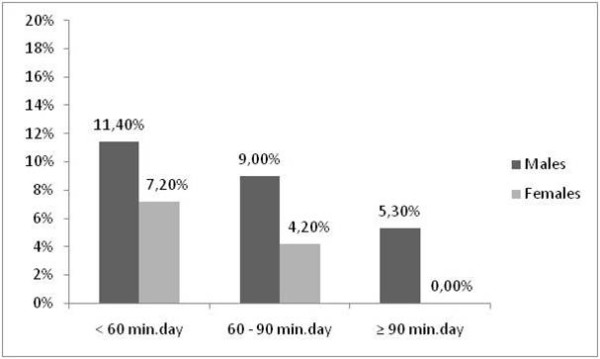
**Prevalence of metabolic syndrome according to time participation in moderate-to-vigorous physical activity**. *p < 0.05 between the physical activity levels (Chi-squared test).

**Figure 2:**
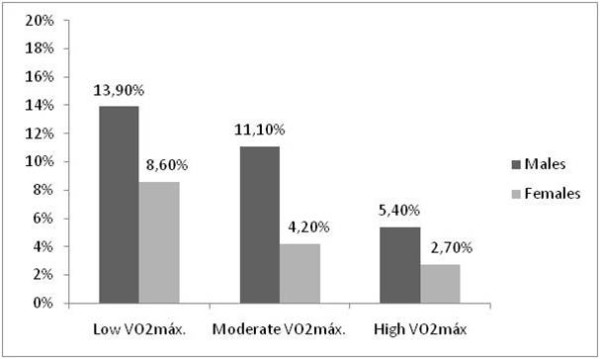
**Prevalence of metabolic syndrome according to cardiorespiratory fitness levels**. *p < 0.05 between the cardiorespiratory fitness levels (Chi-squared test).

The prevalence of MetS by physical activity and cardiorespiratory fitness levels is shown in Figures 1 and 2. A higher prevalence of MetS was observed in the inactive adolescents and in the adolescents with low cardiorespiratory fitness (p < 0.05); there was no difference with respect to gender. Significant associations were demonstrated between MetS and cardiorespiratory fitness (Table 3). The subjects with low cardiorespiratory fitness had an odds ratio for MetS of 3 (CI, 1.13-7.94) compared with subjects with high cardiorespiratory fitness. No significant association was observed between MetS and physical activity.

**Table 3:** Logistic regression of cardiorespiratory fitness and physical activity levels with the metabolic syndrome in Brazilian adolescents

		Metabolic syndrome OR (95% IC)	
	
	N	non-adjusted	adjusted for age and gender
High VO_2máx _(3^rd ^tertile)	152	1	1
Moderate VO_2máx_. (2^nd ^tertile)	154	1.96 (0.64-7.08)	1.9 (0.70-5.48)
Low VO_2máx_. (1^st ^tertile)	150	3.32* (1.16-7.08)	3.0* (1.13-7.94)
Very active (≥ 90 min/day of MVPA)	56	1	1
Active (≥ 60 and < 90 min/day of MVPA)	217	1.77 (0.20-5.68)	1.15 (0.56-2.37)
Inactive (< 60 min/day of MVPA)	183	2.32 (0.29-8.58)	1.25 (0.60-2.60)

* p < 0.05 (Wald test)

Abbreviations: OR, odds ratio; 95% IC, Confidence interval of 95%.

## Discussion

Cardiovascular disease risk factors in children and adolescents have been assessed in many studies. Most studies have looked at a single or a combination of risk factors [22-25]. Several studies have also used diverse definitions of pediatric MetS [4,26,27]. There are few studies pertaining to the prevalence of MetS in Brazilian children and adolescents with which to compare. Our data showed that the prevalence of MetS in Brazilian adolescents (7.7%) is consistent with previously published studies. Data from these studies suggest that the prevalence of MetS varies between 3% and 12% in a representative sample of youth [4,15,16,28].

Rodrigues et al. [29] evaluated 380 subjects (Vitoria, ES, Brazil) and reported a prevalence of MetS in 1.3% of adolescents. Cavali et al. [30] evaluated 80 obese adolescents (São Paulo, SP, Brazil) and reported a prevalence of 13.7% using the International Diabetes Federation criteria and 15% by Jolliffe and Janssen criteria. These studies highlight the need for standardized criteria to diagnose MetS in children and adolescents. Therefore, findings using different diagnostic criteria for MetS should be made with caution.

Data from the NHANES 1999-2002 showed that adolescents with the lowest physical activity level had a significantly increased prevalence of MetS in both genders [31]. In our study, the associations between physical activity and MetS were not significant. This was the case even when we used a continuous score to define MetS (Data not shown). Nevertheless, determining the MetS prevalence by physical activity and cardiorespiratory fitness suggest that adolescents with higher physical activity levels and cardiorespiratory fitness had lower MetS prevalence. Similarly, in the study by Nguyen et al. [32], the odds of MetS among youth in the lowest physical activity group (< 43 min of physical activity per day) were five times greater than those in the highest physical activity group (> 103 min per day). Conversely, Pan and Pratt [33] examined the association of MetS with physical activity in 4,450 US adolescents and demonstrated that despite the tendency of a lower prevalence of MetS in active subjects, this association was not statistically significant.

In terms of cardiorespiratory fitness, the associations reported in the present study are supported by previous research. Andersen et al. [34] reported a lower VO_2max _in adolescents with three or more risk factors. A recent study [35] demonstrated that unfit Azorean Islands adolescents were more likely (OR, 3.414; 95% CI, 1.150-10.129) to be diagnosed with MetS when compared to the fittest adolescents.

Therefore, the evidence suggests that physical activity and cardiorespiratory fitness are important indicators of the risks for developing MetS [34]. Thus, pediatric researchers have investigated how these individual lifestyle components increase the metabolic risk from childhood and adolescence to adulthood [36-38].

In a review of the secular trends in variables associated with the MetS of North American youth, Eisenmann [39] showed that cardiorespiratory fitness and physical activity has not changed in youth in recent decades. McMurray et al. [40] reported a high incidence of MetS in adolescents with a history of low fitness and physical activity. Steele et al. [41] suggested that physical activity and cardiorespiratory fitness influence metabolic risk trough separate pathways. An important distinction between physical activity and cardiorespiratory fitness is intra-individual day-to-day variability; physical activity will undoubtedly vary on a daily basis, whereas cardiorespiratory fitness will remain relatively static, taking time to change. This variability impacts the ability to measure these two variables and consequently influences their relationship with metabolic outcomes [41].

Further, it has been difficult to argue for specific levels of physical activity or fitness in children and adolescents, which could indicate an unhealthy condition [41-43]. However, the optimal amounts of physical activity and cardiorespiratory fitness required for preventing and treating the MetS in children and adolescents is unknown. Therefore, we propose substituting part of the sedentary time with light physical activity, then advancing to moderate-intensity, increasing goals and tailored on individual characteristics, up to an end point represented by daily moderate-to-vigorous intensity program that is sufficient to achieve normalization of the metabolic profile [41-43].

The primary limitation of this study was that MetS outcomes were dependent on our definition of MetS, a problem inherent to the lack of consensus about the criteria for diagnosing MetS in a pediatric population. In addition, the cross-sectional study design does not guarantee the temporal precedence of variables and limit the extrapolation of observations. Another limitation of the study was the use of self-report instruments to measure physical activity. However, the physical activity record is considered a valid instrument for physical activity evaluation [19] and is widely used in studies involving young people [44-47]. A potential factor that may indirectly influence MetS prevalence is socioeconomic status, which was not evaluated in this sample. Therefore, future studies should use socioeconomic status as a control variable when assessing the influence of physical activity and cardiorespiratory fitness on risk of MetS.

Future research should also examine what is the optimal amount of physical activity needed to maximize metabolic health benefits in children and adolescents. In order to facilitate this examination, a standardization of the MetS definition should be further discussed among organizations and experts in the field. Finally, future studies should use both dichotomous and continuous scoring for MetS. In our study, continuous scoring did not result in stronger associations between physical activity and MetS. However, differences in sample size and sample characteristics may result in different associations depending on the selected approach for scoring MetS.

## Conclusion

In summary, our results demonstrate that the prevalence of MetS is higher among Brazilian adolescents who are inactive and have low cardiorespiratory fitness, and there was a significant relationship between MetS and cardiorespiratory fitness. This indicates that long-term prevention strategies should be developed to increase physical activity and cardiorespiratory fitness early in life, with the goal of promoting a physically active lifestyle.

## Abbreviations

MetS: metabolic syndrome; HDL-C: high-density lipoprotein-cholesterol; BP: blood pressure; SBP: systolic blood pressure; DBP: diastolic blood pressure; MVPA: moderate to vigorous physical activity; SD: standard deviation; OR: odds ratio; 95% CI: confidence interval of 95%; ns: non-significant.

## Competing interests

The authors declare that they have no competing interests.

## Authors' contributions

ASN was the principal researcher responsible for the collection, analysis, and interpretation of data, as well as for drafting the manuscript. RDB, AZU, and LPGM were involved in analysis and interpretation of data and also in the critical revision of the paper. MCSB, SGS, WC, and JES were involved in revising the manuscript critically for important intellectual content. All authors read and approved the final manuscript.

## Pre-publication history

The pre-publication history for this paper can be accessed here:

http://www.biomedcentral.com/1471-2458/11/674/prepub
